# A Look Back at an Ongoing Problem: *Shigella dysenteriae* Type 1 Epidemics in Refugee Settings in Central Africa (1993–1995)

**DOI:** 10.1371/journal.pone.0004494

**Published:** 2009-02-13

**Authors:** Solen Kernéis, Philippe J. Guerin, Lorenz von Seidlein, Dominique Legros, Rebecca F. Grais

**Affiliations:** 1 Epicentre, Paris, France; 2 Joint Malaria Programme, Tanga, Tanzania; 3 London School of Hygiene and Tropical Medicine, Wellcome Trust Unit, Bangkok, Thailand; 4 Disease Control in Humanitarian Emergencies, Epidemic and Pandemic Alert and Response, WHO, Geneva, Switzerland; Sabin Vaccine Institute, United States of America

## Abstract

**Background:**

*Shigella dysenteriae* type 1 (*Sd1*) is a cause of major dysentery outbreaks, particularly among children and displaced populations in tropical countries. Although outbreaks continue, the characteristics of such outbreaks have rarely been documented.

Here, we describe the *Sd1* outbreaks occurring between 1993 and 1995 in 11 refugee settlements in Rwanda, Tanzania and Democratic Republic of the Congo (DRC). We also explored the links between the different types of the camps and the magnitude of the outbreaks.

**Methodology/Principal Findings:**

Number of cases of bloody diarrhea and deaths were collected on a weekly basis in 11 refugee camps, and analyzed retrospectively.

Between November 1993 and February 1995, 181,921 cases of bloody diarrhea were reported. Attack rates ranged from 6.3% to 39.1% and case fatality ratios (CFRs) from 1.5% to 9.0% (available for 5 camps). The CFRs were higher in children under age 5. In Tanzania where the response was rapidly deployed, the mean attack rate was lower than in camps in the region of Goma without an immediate response (13.3% versus 32.1% respectively).

**Conclusions/Significance:**

This description, and the areas where data is missing, highlight both the importance of collecting data in future epidemics, difficulties in documenting outbreaks occurring in complex emergencies and most importantly, the need to assure that minimal requirements are met.

## Introduction

*Shigella dysenteriae* serotype 1 (*Sd1*) has been identified as the cause of large-scale dysentery outbreaks, which are often associated with high attack rates (AR) and case fatality ratios (CFRs) [1]–[5]. Over the last fifty years, *Sd1* outbreaks were reported from Central America [6], [7], Bangladesh [8], South Asia [9], and Central [10]–[12] and Southern Africa [13]–[14]. The last reported large-scale *Sd1* outbreak occurred in Sierra Leone in 1999 [15]–[16]. Displaced populations, faced with an insufficient supply of clean water, poor sanitation, overcrowding and concomitant malnutrition are at high risk for dysentery outbreaks [17]–[24].

In 1993 and 1994, civil wars devastated Burundi and Rwanda and led to the displacement of millions of people. In April 1994, approximately 500,000 refugees from Rwanda crossed the border into Tanzania [20]. More than fifteen non-governmental organizations (NGOs) and the United Nations High Commissioner for Refugees (UNHCR) provided medical support. Within days, camps were supplied with drinking and potable water, non-perishable food, latrines and transportation systems [25].

A second mass influx of refugees occurred between July 14 and 17, 1994 when approximately one million people crossed into neighboring Zaire (now Democratic Republic of Congo, DRC). Refugees entered DRC principally through the town of Goma, north of Lake Kivu, and during the following weeks, thousands died of cholera around Goma [20]. Many of the survivors moved further north into DRC to large camps or within the town of Goma. The context in which the camps were set-up in DRC differed substantially from those in Tanzania. In DRC, NGOs were overwhelmed by the massive influx of refugees over a very short time. Water resources, sanitation and local infrastructure were severely deficient [20], [26].

The medical NGO Médecins sans Frontières (MSF) was involved in the response to 11 outbreaks of bloody diarrhea occurring in camps located in Tanzania, Rwanda and DRC, all related to laboratory-confirmed *Sd1*. Here, we use MSF surveillance data to describe the *Sd1* outbreaks in these settlements.

## Methods

We describe 11 outbreaks of bloody diarrhea, which occurred in refugee settings supported by MSF between 1993 and 1995 in Rwanda (Kaduha, Rukondo and Nzangwa), Tanzania (Benaco and Lumashi), and DRC (Kibumba, Mungunga, Katale, Kashusha, Kalehe and Inera).

### Data collection and assumptions

Data presented here come from two different sources: published literature referenced in Pubmed/Medline; and grey literature from the MSF archives, mainly “field reports” written at the time. For each camp, we collected data on (i) number of cases/deaths of bloody diarrhea; (ii) results of bacteriological examinations; and (iii) crowding, sanitation, water and food supply. Bacteriological examinations were not routinely available for individual diagnosis and dysentery was diagnosed clinically. We used the WHO clinical case definition used at the time, to define a dysentery case as any person with diarrhea (passage of 3 or more watery or loose stools in the past 24 hours) and visible blood in the stool [27]. *Sd1* was identified as the causative organism of bloody diarrhea in all eleven camps [19], [20], [28], [29 and MSF unpublished data] and dysentery cases were assumed to be caused by *Sd1*.

In most refugee settings, the surveillance began on August 1994 except for Nzangwa in Rwanda (November 1993) and Benaco and Lumashi in Tanzania (April 1994). As recommended by WHO guidelines [27], the number of cases and deaths related to dysentery were collected on a weekly basis for two age groups: children under five years, and persons five years or older. The mean weekly camp populations (under 5's and 5 years and older) were used as denominator to estimate attack rates (AR). For three camps (Kaduha, Nzangwa and Katale), population data were not available on a weekly basis. In each of these camps, total population size was based on information provided by MSF field workers (based on epidemiologic studies), and was considered constant over time [MSF unpublished data, 24]. The mean weekly population for each camp is shown in Table 1.

**Table 1:** Total population, attack rates and case fatality ratios in 11 refugee settings in Rwanda, Tanzania, and Democratic Republic of Congo between November 1993 and February 1995.

Country	Camp	Arrival of refugees	First cases of bloody diarrhea	Weekly population (Mean±sd)		Attack Rates (%)		Case Fatality Ratios (%)	
				Total	Under 5	Total	Under 5	Total	Under 5
Rwanda	Nzangwa	October 93	November 93	18,930^a^	3,070^a^	30.7	53.2	NA^b^	NA^b^
Tanzania	Benaco	April 94	May 94	215,889±31,856	45,160^a^	15.1	18.9	NA^b^	NA^b^
	Lumashi	June 94	June 94	61,620±21,777	15,600^a^	9.4	13.3	NA^b^	NA^b^
Rwanda	Kaduha	April–July 94	August 94	30,000^a^	NA^b^	12.3	NA^b^	6.0	11.5
	Rukondo	April–July 94	August 94	45,290±13,135	7,682±2,248	18.1	26.2	1.5	1.6
DRC^c^	Mungunga	July 94	August 94	131,900±11,371	22,420±1,933	22.9	28.7	NA^b^	NA^b^
	Kibumba	July 94	August 94	128,800±24,115	21,890±4,099	39.1	49.7	NA^b^	NA^b^
	Katale	July 94	August 94	110,000^a^	18,370^a^	34.4	44.6	NA^b^	NA^b^
	Kalehe	July 94	August 94	8,588±2,227	1,534±400	10.5	17.3	3.8	7.9
	Kashusha	July 94	August 94	36,520±4,730	6,574±851	5.5	5.9	1.9	3.1
	Inera	July 94	September 94	29,080±2,452	5,234±441	6.3	5.0	9.0	18.3

a Was considered constant over time.

b NA: Not Available.

c DRC: Democratic Republic of the Congo.

### Data analysis

Camps were grouped according to the SPHERE requirements for (i) water supply (estimated by the average quantity of water available per person per day); (ii) food availability (estimated by the average food ration in kcal/person/day); and (iii) sanitation (estimated by the average number of individuals using each latrine) [30]. Minimum requirements were 7–15 liters of water /person/day, 2100 kcal/person/day, and a maximum of 20 persons per latrine. For shelter and settlement, health infrastructures and transportation system, precise quantitative estimates were impossible.

As, we could get only an overall picture of the logistic organization and health system in each camp, we considered the size (maximum population) of camps as a proxy classification for complexity and logistic difficulties. We categorized camps as: small (maximum weekly population ranging from 10,000 to 34,000), medium (35,000 to 79,000), and large (80,000 to 217,000). Cutoffs were chosen according to the values of first and third quartile of maximum weekly population. Descriptive statistics were performed using the R statistical package (R Development Core Team; R Foundation for Statistical Computing [http://www.R-project.org]). All data analysed in this work are strictly anonymous and come from a routine surveillance system, therefore no ethical approval was required.

## Results

### Population in camps

The mean weekly population in each camp ranged from 8,588 refugees in the smallest camp of Kalehe (DRC) to 215,889 in the largest (Benaco – Tanzania, table 1). The mean proportion of population under 5 was around 18% (range: 17%–21%). Estimates of water and food supply, number of residents per latrine and proportion of *Sd1* strains resistant to nalidixic acid are shown in table 2.

**Table 2:** Attack rates of bloody diarrhea, water and food supply, sanitation, and resistance to Nalidixic Acid of *sd1* strains in the eleven camps considered.

Country	Camp	Attack rates	Liters of water per person per day^a^	Number of residents per latrine^a^	Food supply^a^ (kcal / person/ day)	Proportion of Sd1 strains resistant to Nalidixic Acid^a^
DRC (Bukavu)	Kashusha	6%	**2**		2000	Close to 100% (N = 20)^b^
DRC (Bukavu)	Inera	6%				
Tanzania	Lumashi	9%	15	20		35%^c^
DRC (Bukavu)	Kalehe	11%	6			Close to 100%^b^
Rwanda	Kaduha	12%	**3**	**200**		97,5%
Tanzania	Benaco	15%	**3.7**			35%^c^
Rwanda	Rukundo	18%			1800	97,5%
DRC (Goma)	Mugunga	23%	**1** d	**1 029**	**800**	100% (N = 38)
Rwanda	Nzangwa	31%	20	**60–120**	**1400**	50% (N = 7)
DRC (Goma)	Katale	34%	**1** d	**184**	**800**	100% (N = 38)
DRC (Goma)	Kibumba	39%	**1** d	**500**	**800**	

Sources: Authors' unpublished data, [19], [29]–[31].

Note: Camps are ordered according to the attack rates of bloody diarrhea. Bold numbers indicate values under SPHERE minimum requirements (water supply<6 L/p/d, food supply<2000 kcal/p/d, number of resident per latrine >20).

a Evaluated at the beginning of the epidemic.

b 20 strains collected in Goma and Bukavu, most of them being resistant to nalidixic acid (no proportions provided by the authors).

c Reached 60% after a few weeks.

d 0.5 Liters at the arrival of refugees, 5 Liters after 11 days.

**Figure 1:**
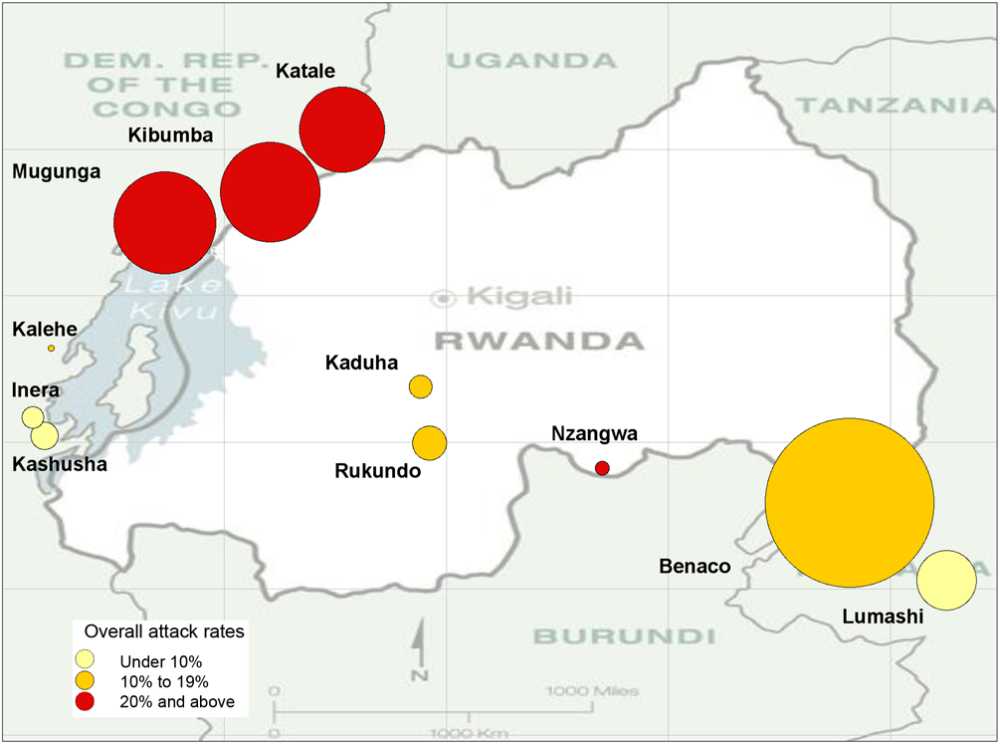
Size of camps and attack rates. Map of the Great Lakes region showing: (1) the size of camps (proportional to the size of circles), and (2) the attack rates (color of circles) between November 1993 and February 1995.

Between November 1993 and February 1995, a total of 181,921 cases of dysentery were recorded. The attack rates varied greatly between camps, ranging from 6% in Inera to 39% in the large camp of Kibumba in DRC (Figure 1). The CFRs for dysentery could be estimated only for five camps. The highest CFR, 9.0% was recorded in Inera (DRC). In the other four camps, the CFRs were lower (Table 1). The CFRs were higher in children under 5 with the highest CFR seen in Inera (18.3%) and lowest CFR (1.6%) in Rukondo. The median duration of dysentery epidemics was 27 weeks, ranging from 5 (in Inera) to 29 weeks in Benaco.

**Figure 2:**
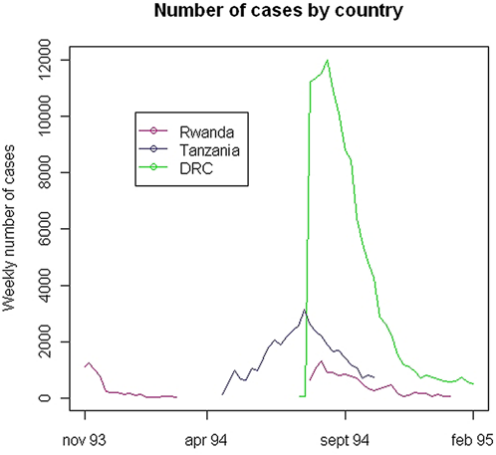
Weekly number of cases of bloody diarrhea between November 1993 and February 1995. The first documented Sd1 epidemic started in Rwanda in Nzangwa camp in November 1993. The epidemic ended in March–April 1994 as Burundian refugees began to return from Rwanda to Burundi at the beginning of the conflict in Rwanda. The two following outbreaks occurred in Tanzania, in Benaco (May–October, 1994) and in Lumashi (June–October, 1994), corresponding to the first massive influx of refugees who fled Rwanda. The major epidemic started in DRC (Democratic Republic of Congo, formerly Zaire) around July–August 1994 and lasted until February 1995. At the same time, a revival of epidemic activity was observed in the Rwandan camps of Kaduha and Rukondo.

**Figure 3:**
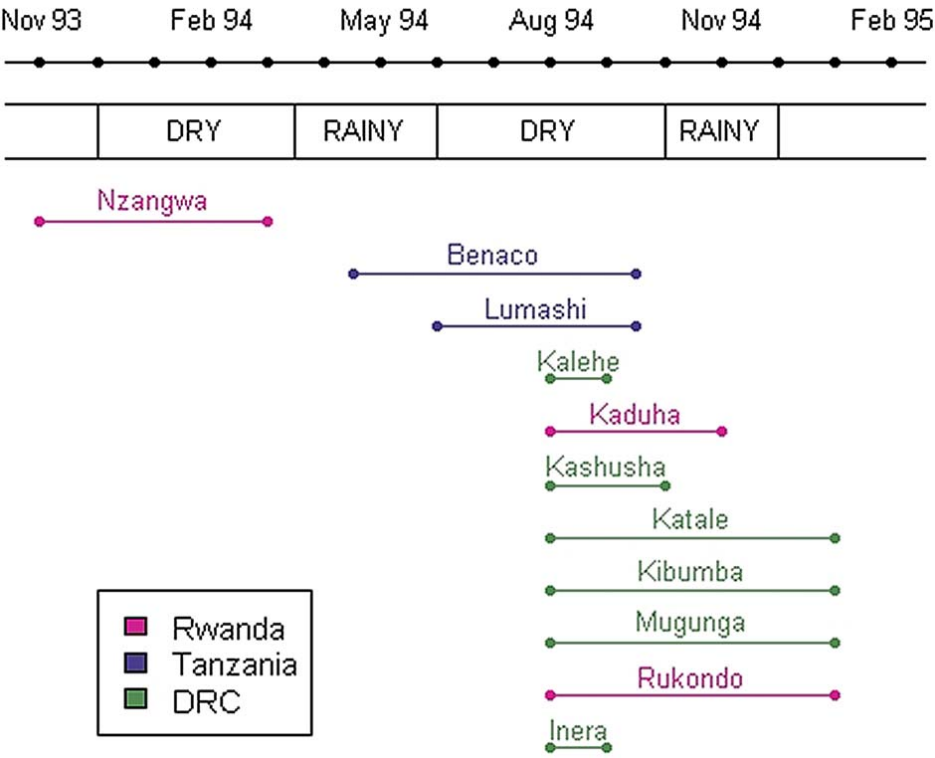
Time course of the epidemics in relation to the seasons. Four seasons are usually observed in the Great Lakes Region: two dry seasons (December to Mid-March and June to October) and 2 rainy seasons (October to December and Mid-March to the end of May). Three outbreaks (in Nzangwa, Benaco and Lumashi) started during the rainy season (November, May and June, respectively) and peaked in the dry season. The start of eight subsequent outbreaks occurred during the dry period. However, since surveillance data was not available from the start of these outbreaks, they are likely to have started at the end of the rainy/beginning of the dry period. Most outbreaks were concentrated between June and October.

The first documented *Sd1* epidemic started in Rwandan camp of Nzangwa in November 1993 (Figure 2). The epidemic ended by April 1994 as the camp residents, Burundian refugees, began to return to Burundi with the onset of the conflict in Rwanda. The next two epidemics occurred in Tanzania, in Benaco (May–October, 1994) and in Lumashi (June–October, 1994), following massive influx of refugees from Rwanda. The largest epidemic started in DRC in August 1994 and lasted until February 1995. At the same time, dysentery outbreaks were reported from the Rwandan camps of Kaduha and Rukondo. Outbreaks occurred throughout the calendar year with eight outbreaks beginning between June and October, during the dry season (Figure 3).

### Differences between camps

The mean attack rates were 18.3% (±9.9), 13.5% (±4.8) and 28.1% (±9.4) in small, medium and large camps respectively (median 12.3%, 10.8% and 34.4%, respectively).

Considering the context of settlement and availability of resources (Table 2), three different situations occurred: (1) Benaco and Lumashi in Tanzania, (2) Goma in DRC, and (3) Bukavu in DRC. In Tanzania, the two camps were supplied with medical support, drinking and potable water, non-perishable food, latrines and transportation systems from the first days of the influx of refugees. Lumashi residents were provided with 15 L of water per day and each latrine was shared by twenty refugees. In Benaco, water supply was inadequate (3.7 L per person per day) at the beginning of the epidemic, but rapidly increased in the following weeks (Table 2). Conversely, in the three camps located around Goma (Katale, Kibumba and Lumashi), the capacity of relief organizations was immediately overwhelmed, the construction of latrines seriously impeded by the rocky volcanic topography of the site, and water resources were severely deficient. In the first days of the epidemic, less than 1 L of water was available per person and per day, and hardly reached 3 L after 6 days. In these camps, these estimates correspond to the amount of “safe” water delivered by NGOs. Refugees completed their daily water ration by their own means (streams, lake, etc.). The number of residents per latrine was extremely high, from 184 per latrine in Katale to 1030 in Mugunga. As expected, the mean attack rate was lower in the camps with favorable context of settlement, compared to those where relief capacities were quickly overwhelmed, in the region of Goma (13.3%±2.1 versus 32.1%±6.9 respectively). In the region of Bukavu (South of Lake Kivu, DRC), attack rates were much lower than in Goma. Delivered water supply reached 2 to 6 L per resident per day. The average food ration, although under the minimum recommended, was the highest among the eleven camps considered (2000 kcal/person/day in Kashusha). The three camps in Bukavu located in this region reported good organization of the sites, with shelters available for all refugees, laid out in lines and rows, and the availability of medical support.

## Discussion

Civil disturbances in 1993–1995 in the Great Lakes region led to large-scale and prolonged epidemics of *Sd1*, with major burden among displaced populations. The observed CFRs were higher in children under 5, confirming previous observations [17]–[19]. As expected, the context of settlement, availability of resources and speed of response seemed to be crucial to reduce the burden of *Sd1* outbreaks, as noted when examining the mean attack rates: 13.3% (standard deviation, sd = 2.1) in Tanzania, where resources were quickly available versus 32.1% (sd = 6.9) in the region of Goma.

Data presented are based on a clinical definition of dysentery from a surveillance system put in place in the context of a civil war and major humanitarian crisis. In this context, like in many complex emergencies, the surveillance system was the only source of data available and confirmation of cases was not possible. Although attempts were made to derive a model for attack rates, we feel that the data were too disparate to identify explanatory factors by statistical modelling, and chose to present a descriptive analysis. This surveillance data does not reflect all *Shigella* cases, but rather provides a guide concerning the magnitude of the outbreaks. Previous studies suggest that less than one-third of culture-proven shigellosis episodes present with dysentery [3].

Special attempts were made to obtain precise and reliable data from all camps in the region, but we nevertheless made several assumptions. For example, the count of the total population was not available on a weekly basis in all camps (Kaduha, Nzangwa and Katale). However, population estimates in the 8 other camps (based on weekly estimations) were relatively constant over time, and estimates provided by MSF field workers (based on epidemiologic studies) were concordant with the published literature. Despite intensive literature search performed both in Pubmed and the archives of MSF, key indicators could not be estimated for each camp. Despite the need to make assumptions about missing data, this description is consistent with those presented in previous observational studies in terms of attack rates and case fatality ratios [19]–[24].

The CFRs observed during this crisis were high compared to the mortality found in recent epidemiological studies in endemic settings in Asia [3]. These differences can be related to host factors such as nutrition status, co-infections or the virulence of the organism including the emergence of antibiotic resistances in Central Africa at that time. The strain identified in the region of Goma (DRC) was resistant to most commonly used antibiotics, including nalidixic acid [20]. Inappropriate antibiotic treatment likely played a significant role in the emergence of resistance, and consequently on the CFRs. These data likely underestimate the true CFR, especially in a context of high resistance [32]. We present crude data from number of sources, which provide only snapshots of resistance. Ideally, microbiologic examination should have been performed throughout the duration of the outbreaks which was not possible in an emergency setting. Where data were available, we examined the weekly CFR by week and no particular trend was observed, however it is probable that conjunction of antibiotic resistance, poor sanitary conditions and insufficient food and water supply lead to the major crisis observed in Goma.

There may well be a place for a future *Shigella* vaccine which would be of great interest in the prevention of dysentery outbreaks and containment of drug resistance. Such vaccine could potentially be used in a reactive way, although preventive use is clearly preferable. The utility of a future vaccine would largely depend on its administration (e.g. number of injections, oral or injectable). Meanwhile, effective antibiotics have to be readily available where needed. Considering the current resistance data available, quinolone II generation (e.g. ciprofloxacin) is now recommended [33].

This description, and the areas where data is missing, highlight both the importance of collecting data in future epidemics and the potential difficulties in documenting outbreaks occurring in complex emergencies. The contrast between camps located in Tanzania compared to those in the region of Goma (DRC), in terms of number of cases and deaths related to bloody diarrhoea, further reinforce the need for a rapid response by health agencies. The top ten public health priorities for complex humanitarian emergencies [34], [35] should be kept in mind (Box S1).

In 2008, the confrontation between the Congolese Army and an armed opposition group forced 400,000 people from their homes and threatens to cause another major crisis among displaced populations (source: UNHCR). If *Sd1* is still circulating in the region, it could re-emerge and have a devastating effect.

## Supporting Information

Box S1 Top Priorities to Address in Emergencies [34], [35]
(0.02 MB DOC)
