# Supplementary material for: A Look Back at an Ongoing Problem: Shigella dysenteriae Type 1 Epidemics in Refugee Settings in Central Africa (1993–1995)
Source: PLoS One. 2009 Feb 13;4(2):e4494. doi: 10.1371/journal.pone.0004494 (PMC2636862; doi:10.1371/journal.pone.0004494)
Supplement: Box S1 — Top Priorities to Address in Emergencies [34], [35] (0.02 MB DOC) [file pone.0004494.s001.doc]

Box S1: Top Priorities to Address in Emergencies [34, 35]

- Rapid assessment of the health status of the population
- Mass vaccination against measles
- Water supply and implementation of sanitary measures
- Food supply and implementation of specialized nutritional rehabilitation programs
- Shelter, site planning, and non-food items
- Curative care based on the use of standardized therapeutic protocols, using essentials drugs
- Control and prevention of communicable diseases and potential epidemics
- Surveillance and alert
- Assessment of human resources and training and supervision of community health workers
- Coordination of different operational partners
